# COVID-19 and related social distancing measures induce significant metabolic complications without prominent weight gain in Korean adults

**DOI:** 10.3389/fmed.2022.951793

**Published:** 2022-09-07

**Authors:** Hae-Jin Ko, Yoon Jeong Cho, Kyoung-Kon Kim, Jee-Hyun Kang, Young-Sang Kim, Ji-Hee Haam, Yang-Im Hur, Hye-In Choi, Kyu Rae Lee, Jung Ha Park, Soo Hyun Cho, Jong-Koo Kim, Taesic Lee, Myung-Jae Seo, Yeong Sook Yoon, Yoobin Seo, Ga Eun Nam, Sun Hyun Kim

**Affiliations:** ^1^Department of Family Medicine, School of Medicine, Kyungpook National University Hospital, Daegu, South Korea; ^2^Department Family Medicine, Daegu Catholic University School of Medicine, Daegu, South Korea; ^3^Department of Family Medicine, Gachon University College of Medicine, Gachon University Gil Medical Center, Incheon, South Korea; ^4^Department of Family Medicine, Konyang University College of Medicine, Daejeon, South Korea; ^5^Department of Family Medicine, CHA Bundang Medical Center, CHA University, Seongnam, South Korea; ^6^Chaum Life Center, CHA University, Seoul, South Korea; ^7^Department of Family Medicine, Jeju National University Hospital, Jeju, South Korea; ^8^Department of Family Medicine, College of Medicine, Chung-Ang University Hospital, Seoul, South Korea; ^9^Department of Family Medicine, Yonsei University Wonju College of Medicine, Wonju-si, South Korea; ^10^Department of Family Medicine, Inje University Ilsan Paik Hospital, Goyang-si, South Korea; ^11^Department of Family Medicine, Sanbon Medical Center, School of Medicine, Wonkwang University, Gunpo-si, South Korea; ^12^Department of Family Medicine, Korea University Guro Hospital, Korea University College of Medicine, Seoul, South Korea; ^13^Department of Family Medicine, International St. Mary's Hospital, Catholic Kwandong University College of Medicine, Incheon, South Korea

**Keywords:** body weight changes, COVID-19, metabolic syndrome, obesity, physical distancing, weight gain

## Abstract

**Background:**

This study using multi-center health examination data from Korean adults was conducted to confirm changes in weight, and their related cardiometabolic parameters, before and after strengthening of social distancing regulations.

**Methods:**

A retrospective cohort study was conducted using health check-up data from 13 university hospitals. The study period was from January 2018 to July 2020. To examine the effect of systematic social distancing measures, participants who underwent a health check-up (Visit 3) between July 2020 and July 2021 (during full scale social distancing), and had undergone two previous health check-ups (Visits 1 and 2) between January 2018 and June 2020 (before social distancing), were selected. In total, data from 7,875 participants were analyzed. Linear mixed-effect models were used to calculate estimates of anthropometric indices and metabolic markers measured on Visits 2 and 3, compared with measurements from Visit 1.

**Results:**

There were no significant differences in body weight, body mass index, waist circumference, and body composition on Visit 3 than on Visits 1 and 2. However, the odds of metabolic syndrome and its components, including hypertension, high glucose, diabetes, hypercholesterolemia, hypertriglyceridemia, hyper-non-high-density lipoprotein cholesterolemia, and dyslipidemia were significantly higher on Visit 3 than on Visits 1 and 2. The increase in metabolic complications was marked, particularly in relatively young adults who visited health check-up centers located in the capital area.

**Conclusion:**

Metabolic syndrome and its components were significantly worse after high level social distancing, although there were no significant increases in anthropometric indices and body fat levels. Healthcare providers need to prevent and manage worsening of metabolic parameters in subpopulations prone to be more sedentary and eat unhealthy food during the COVID-19 pandemic and associated social distancing measures.

## Introduction

On March 11th, 2020, the World Health Organization declared a pandemic of Severe Acute Respiratory Syndrome coronavirus 2 (SARS-CoV-2) disease (COVID-19) (1). Since then, more than half a billion people have been infected, and 6 million have died worldwide; as of May 3rd, 2022, there have been 17.3 million confirmed cases and 23,000 deaths in South Korea (2). The whole world is suffering from the physical and psychological damage, as well as economic burden, of the pandemic.

**Figure 1:**
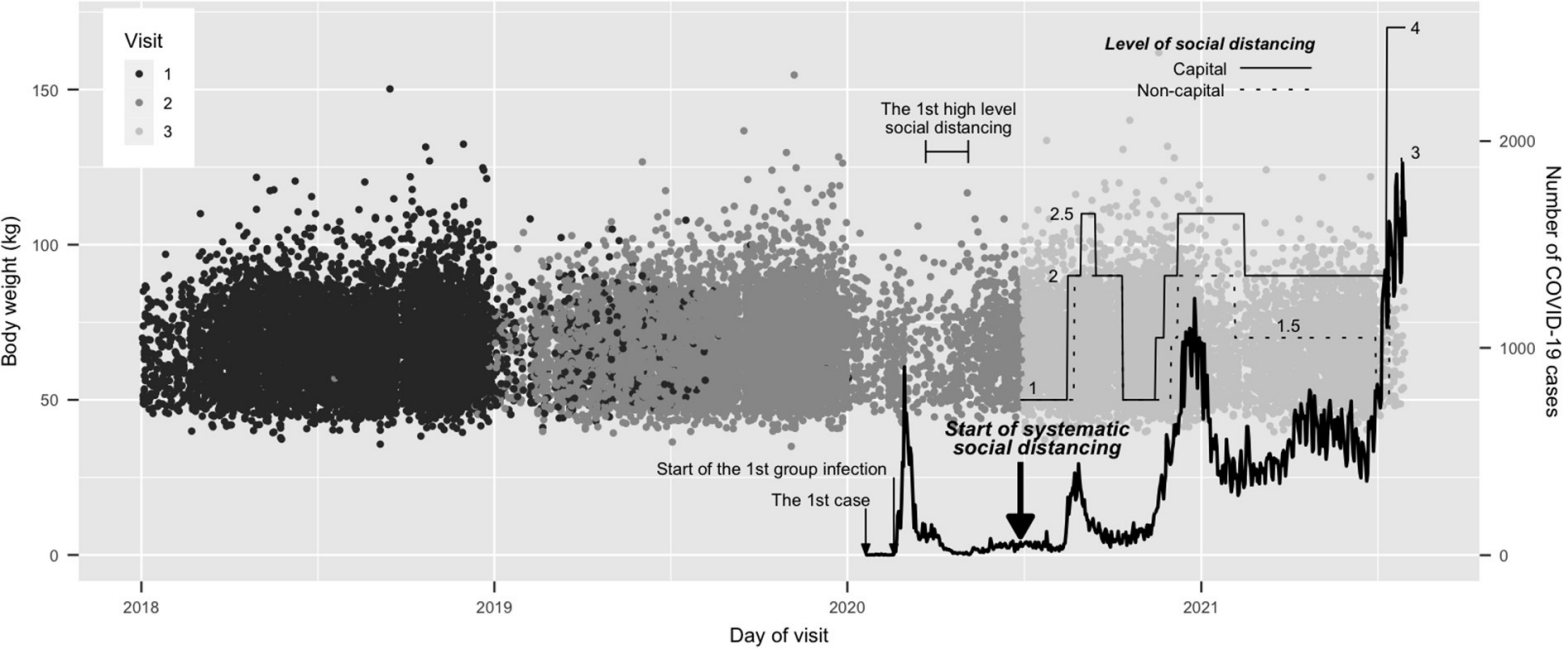
Body weight on each visit day, along with the daily case numbers of COVID-19 and the level of social distancing. All final visits occurred after the start of the systematic social distancing, and all the first visits were before that. The levels of social distancing in capital and non-capital areas are shown, with the final four-level system. The solid line denotes a capital area and the dotted line denotes a non-capital area. The levels of social distancing in capital areas were always the same or higher than those in non-capital area.

In 2020, to cope with the rapid spread of COVID-19, the Korean Ministry of Health and Welfare introduced a social distancing policy; meanwhile, several countries, including European countries, the United States, and Canada imposed social distancing and lockdowns. Daily numbers of new COVID-19 cases and levels of social distancing are illustrated in Figure 1. In South Korea, the first COVID-19 patient was diagnosed on January 20th, 2020, and the first wave of COVID-19 began with the first large-scale group infection on February 18th, 2020. On February 29th, 2020, the Korean Government began recommending that people maintain a distance of least 2 meters from each other, and on March 22, 2020, announced strict social distancing measures that included closure of churches, clubs, and gyms. As the number of new daily cases of COVID-19 dropped to single digits, the high level social distancing policy was relaxed on April 20th, 2020. Social distancing was further downgraded on May 6th, 2020 in accordance with the concept of “social distancing in daily life.” On June 28th, 2020, the Korean Government introduced a more detailed and systematic social distancing system. However, a spike of new cases in August 2020 led the health authorities to upgrade the social distancing level temporarily. With the start of 3rd wave in late November 2020, the government upgraded the regulations again and maintained high level social distancing for several months. The regulations recommended staying at home and restricting private gatherings (no more than five people), mass gatherings (schools, sports events, films, or musical shows), and the usage of multi-person facilities, including fitness centers; these rules were maintained until May 2021 (2).

Social distancing plays a vital role in preventing the spread of COVID-19 (3, 4); however, its side effects include psychiatric problems such as depression, anxiety, and pain, along with a decreased social activity (5–7). Moreover, social distancing has restricted and reduced the use of sports facilities such as fitness centers and swimming pools, and the number of days of school attendance, resulting in a sharp reduction in physical activity. Several studies report that physical activity had decreased (8, 9), whereas physical symptoms have increased after lockdown (10). In addition, the social distancing decreased the number of time people ate out and increased the delivery and consumption of high calorie foods (11).

Thus, it is possible to predict that social distancing will be associated with weight gain or deterioration of metabolic markers such as blood sugar and lipid profiles. Several studies report significant weight gain or worsening of cardiometabolic parameters during the COVID-19 pandemic (12–15), while a few report a contrary result (16) or both results (17, 18). Previous studies report controversial results because they were based on short-term follow-up data, mostly from questionnaires, leading to inadequate information on which to base an assessment of fundamental weight changes and related parameters after lockdown. Furthermore, to the best of our knowledge, no study has examined data from Korean adults obtained during social distancing regulations and after the 3rd wave of COVID-19. Therefore, the aim of this study was to examine multi-center health examination data obtained from Korean adults and investigate changes in weight and related cardiometabolic parameters before and after the start of strengthening the social distancing regulations.

## Materials and methods

### Study design and subjects

This was a retrospective cohort study conducted using health check-up data from university hospitals. Thirteen health check-up centers took part: four in Seoul, two in Gyeonggi Province, two in Incheon, one in Daejeon, two in Daegu, one in Wonju, and one in Jeju.

The study period covered more than 3.5 years, from January 2nd, 2018, to July 30^th^, 2020. Figure 1 shows body weight and visit day, along with the daily case numbers of COVID-19 and the level of social distancing. Based on this, the study aimed to investigate whether the COVID-19 pandemic and the related strong social distancing measures affected body weight and metabolic parameters. Since June 28, 2020, new systematic social distancing regulations were applied to the whole Korean population; therefore, the study period was divided into two parts, before and after July 2020. All people who underwent health check-ups more than once between July 2020 and July 2021 during full scale social distancing (Visit 3), along with previous health check-ups between January 2018 and June 2020 (i.e., before the social distancing; Visit 1 and Visit 2), were included. The period of the first visit was from January 2nd, 2018, to January 2nd, 2020, before the appearance of COVID-19 in South Korea.

The exclusion criteria were as follows: thyroid disease (thyroiditis, hyperthyroidism, hypothyroidism, serum thyroid stimulating hormone > 10 or <0.01 mg/dL); uncontrolled diabetes (fasting blood glucose > 240 mg/dL or HbA1c > 10%); suspected chronic kidney disease (serum creatinine > 2 mg/dL); active liver disease (serum AST or ALT > 200 mg/dL); liver cirrhosis; a positive anti-HIV antibody test; past history of malignancies; suspected malignancies from the results of endoscopy or abdominal ultrasonography, chest CT, or abdominopelvic CT; bowel preparation for colonoscopy; and prescription of drugs that can cause weight change (e.g., steroids, anti-obesity drugs, sulfonylurea, thiazolidinediones, anti-depressants, and anti-psychotics). The exclusion criteria were confirmed by self-recorded questionnaires, medical records, anthropometric measurements, and the results of laboratory tests, endoscopy, colonoscopy, abdominal ultrasonography, and CT scans. In total, data from 7,875 participants were included in the final dataset.

### Primary and secondary endpoints

The primary surrogate endpoints were the changes in body weight and body mass index (BMI), and changes in body composition indices such as skeletal muscle mass (in kg), body fat mass (in kg), and body fat percentage measured by the bioelectrical impedance analysis (InBody 770 and InBody 970, InBody Co., Ltd, Seoul, South Korea). Secondary surrogate endpoints were changes in waist circumference (WC), blood pressure (BP), serum levels of glucose and lipids, the percentage of HbA1c, and the prevalence of metabolic syndrome and other metabolic complications such as hypertension, diabetes, and dyslipidemia.

### Measurements and definitions

Participants visited the health check-up centers after fasting for more than 12 h. Trained nurses took a past medical history, personal history, and measured anthropometric indices, BP, and pulse rate. Past medical history, including prescription of drugs for hypertension, diabetes, dyslipidemia, and other conditions, was assessed. Age was grouped as ≤ 39, 40–59, and ≥ 60 years. Heavy drinking was defined as alcohol consumption more than 210 g/week for men and 140 g/week for women. Smoking status was classified as non-, ex-, or current according to lifetime exposure to cigarettes. Regarding the location of the health check-up centers, Seoul, Gyeonggi Province, and Incheon were categorized as the capital area, whereas Daegu, Wonju, and Jeju were categorized as non-capital areas.

Height, to the nearest 0.1 cm, was measured in the erect position using a stadiometer. Body weight (measured while wearing a light gown and in bare feet) was measured using digital scales, and WC (to the nearest 0.1 cm) was measured with a tape measure. The anatomical site used for WC measurement was the midpoint between the lower border of the rib cage and the highest point of the iliac crest. With a rest more than 15 min, BP and pulse rate were measured with automatic oscillometric BP devices. Body composition was as described above. Blood samples were taken and complete blood counts, glycosylated hemoglobin percentage, levels of glucose, lipids, aminotransferases, gamma-glutamyl transpeptidase, blood urea nitrogen, creatinine, electrolytes, hepatitis B antigens and antibodies, hepatitis C antibodies, and human immunodeficiency virus antibodies were measured. The glomerular filtration rate was estimated from the CKD-EPI equation.

The definition of metabolic syndrome was based on the National Cholesterol Education Program Adult Treatment Panel III (NCEP-ATP III) consensus definition (19), using Korean-specific cutoff values for WC (20) according to the Korean Society for the Study of Obesity. To fulfill the definition of metabolic syndrome, an individual must have three or more of the following criteria: (i) WC ≥ 90 cm for men; ≥ 85 cm for women, (ii) Triglycerides ≥ 150 mg/dL, or taking dyslipidemia drugs, (iii) HDL cholesterol <45 mg/dL for men; <50 mg/dL for women, (iv) Systolic BP ≥ 130 mmHg or diastolic blood pressure ≥ 85 mmHg, or treatment of previously diagnosed hypertension, (v) Fasting plasma glucose ≥ 100 mg/dL, or treatment of previously diagnosed type 2 diabetes mellitus.

A high WC was defined as ≥ 90 cm for men and ≥ 85 cm for women. High blood pressure was defined as systolic BP ≥ 130 mmHg or diastolic BP ≥ 85 mmHg, or taking hypertension drugs. High serum glucose was defined as fasting glucose ≥ 100 mg/dL or taking diabetes drugs. Hypertension was defined as systolic BP ≥ 140 mmHg or diastolic BP ≥ 90 mmHg, or taking hypertension drugs. Diabetes mellitus was defined as fasting glucose ≥ 126 mg/dL, or HbA1c ≥ 6.5%, or taking diabetes drugs. Hypercholesterolemia was defined as total serum cholesterol ≥ 240 mg/dL, or taking dyslipidemia drugs. Hypertriglyceridemia was defined as serum triglyceride ≥ 200 mg/dL, or taking dyslipidemia drugs. Hypo-HDL cholesterolemia was defined as serum HDL cholesterol <40 mg/dL. Hyper-low density lipoprotein (LDL) cholesterolemia was defined as serum LDL cholesterol ≥ 160 mg/dL or taking dyslipidemia drugs. Hyper-non-HDL cholesterolemia was defined as calculated serum non-HDL cholesterol ≥ 190 mg/dL or taking dyslipidemia drugs. Dyslipidemia was defined as having hypercholesterolemia, hypertriglyceridemia, hypo-HDL cholesterolemia, or hyper-LDL cholesterolemia.

### Statistical analyses

Data are presented as number (percentage) or as the mean ± standard deviation, unless specified otherwise. To compare characteristics between men and women, the Chi-squared test and Student's *t*-test were used. A paired *t*-test was used to compare changes in each variable between the period of Visits 1–2 and that of Visits 2–3. Odds ratios for metabolic syndrome and metabolic comorbidities at each visit were calculated using generalized linear mixed-effect models. The models were adjusted for sex, age group, smoking, and alcohol drinking. The reference for the odds ratio for each visit was Visit 1. All statistical analyses were performed using R version 4.1.2 (R core team, Vienna, Austria). Generalized linear mixed-effect models were performed in lme4 package version 1.1–28, and *post-hoc* analyses were conducted using the Bonferroni method in emmeans package version 1.7.3. A two-tailed *P* < 0.05 was considered statistically significant.

## Results

### Baseline characteristics

The general characteristics of the participants are described in Table 1. The proportion of men and women was roughly equal. More than 70% of the participants were aged 40–59 years. The smoking rate and the percentage of participants who took drugs for hypertension, diabetes, or dyslipidemia were higher for men than for women. The mean BMI was higher in men, while the mean fat percentage was higher in women. More men met the NCEP-ATP III definition of metabolic syndrome. Mean BP, blood glucose levels, HbA1c, triglycerides, and LDL cholesterol levels were higher in men, whereas the HDL cholesterol levels were higher in women. There was no significant difference between the sexes with respect to total cholesterol. The mean duration between visits was 389 ± 97 days between Visits 1 and 2, and 414 ± 115 days between Visits 2 and 3.

**Table 1:** Baseline characteristics of the study population.

	**Men (*N* = 3,960)**	**Women (*N* = 3,915)**	**Total (*N* = 7,875)**	***P*–value**
Age (years)				<0.001
−39	777 (19.62)	935 (23.88)	1,712 (21.74)	
40–59	2,952 (74.55)	2,780 (71.01)	5,732 (72.79)	
60–	231 (5.83)	200 (5.11)	431 (5.47)	
Heavy drinking	2,552 (70.99)	2,058 (58.33)	4,610 (64.72)	<0.001
Smoking				<0.001
Non-smoker	1,713 (46.74)	2,828 (79.89)	4,541 (63.03)	
Ex-smoker	1,085 (29.60)	334 (9.44)	1,419 (19.69)	
Current smoker	867 (23.66)	378 (10.68)	1,245 (17.28)	
Hypertension drug	594 (16.04)	326 (8.91)	920 (12.50)	<0.001
Diabetes drug	195 (5.27)	112 (3.06)	307 (4.17)	<0.001
Dyslipidemia drug	391 (10.56)	296 (8.09)	687 (9.33)	<0.001
Body weight (kg)	72.88 ± 11.50	61.52 ± 11.95	67.23 ± 13.03	<0.001
BMI (kg/m^2^)	24.71 ± 3.13	22.84 ± 3.37	23.78 ± 3.38	<0.001
Waist circumference (cm)	85.92 ± 8.68	79.00 ± 9.34	82.48 ± 9.66	<0.001
Skeletal muscle mass (kg)	31.39 ± 9.17	26.12 ± 7.31	28.63 ± 8.66	<0.001
Fat mass (kg)	18.27 ± 6.08	17.8 ± 5.88	18.04 ± 5.99	<0.001
Fat percentage (%)	24.83 ± 6.01	28.63 ± 6.45	26.71 ± 6.51	<0.001
Metabolic syndrome	914 (24.63)	659 (18.03)	1,573 (21.35)	<0.001
Blood pressure (mmHg)				
Systolic	121.58 ± 12.81	115.35 ± 13.79	118.48 ± 13.66	<0.001
Diastolic	76.47 ± 9.78	72.13 ± 10.29	74.31 ± 10.27	<0.001
Pulse rate (/min)	70.92 ± 11.32	72.81 ± 11.32	71.86 ± 11.36	<0.001
Glucose (mg/dL)	98.17 ± 16.28	92.10 ± 13.5	95.16 ± 15.27	<0.001
HbA1c (%)	5.53 ± 0.58	5.40 ± 0.46	5.47 ± 0.52	<0.001
Total cholesterol (mg/dL)	192.67 ± 34.69	192.40 ± 34.52	192.54 ± 34.61	0.734
Triglyceride (mg/dL)	139.90 ± 88.67	105.89 ± 72.86	122.99 ± 82.96	<0.001
HDL cholesterol (mg/dL)	52.30 ± 13	60.15 ± 14.64	56.20 ± 14.39	<0.001
LDL cholesterol (mg/dL)	122.59 ± 30.93	118.87 ± 30.79	120.74 ± 30.92	<0.001

Data are expressed as N (%) or as the mean ± standard deviation.

P-values are calculated using the Chi-squared test or Student's t-test.

BMI, body mass index; HbA1c, glycated hemoglobin; HDL, high density lipoprotein; LDL, low density lipoprotein.

### Changes in obesity indices at each visit

**Figure 2:**
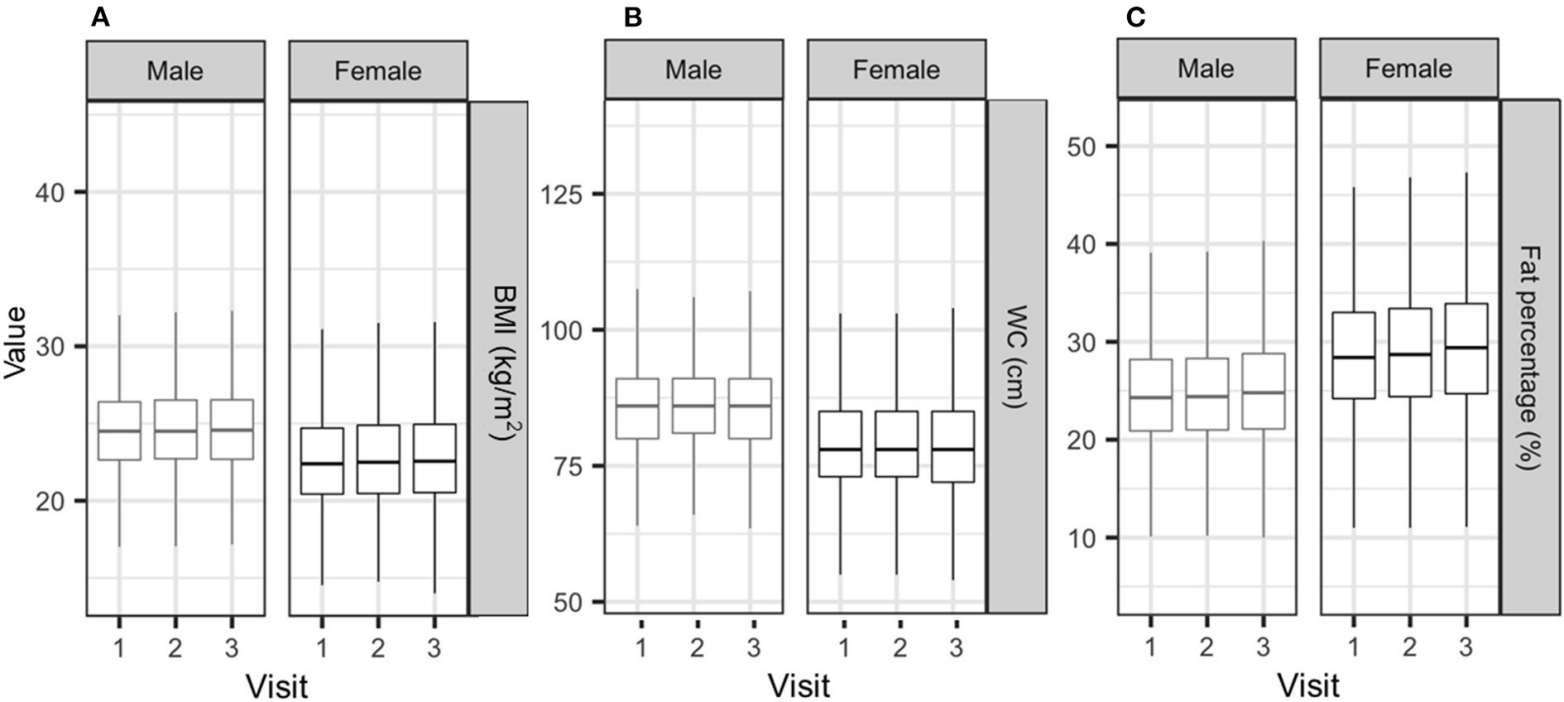
Distribution of BMI **(A)**, waist circumference **(B)**, and body fat percentage **(C)** according to sex. BMI, body mass index; WC, waist circumference.

The distribution of BMI, WC, and body fat percentage is shown in Figure 2. While the size of the changes is too small to be seen in the figures, BMI and body fat percentage increased steadily at each visit, with a decrease in WC at Visit 3. Body weight increased by 0.16 [0.09–0.22] (estimate [95% confidence interval]) and 0.33 [0.26–0.39] kg, and BMI increased by 0.07 [0.05–0.10] and 0.12 [0.09–0.14] kg/m^2^ at Visits 2 and 3, respectively, compared with Visit 1 (*P* < 0.05). WC increased by 0.38 [0.27–0.49] cm at Visit 2 and decreased by 0.19 [0.08–0.30] cm at Visit 3, compared with Visit 1 (P < 0.05). Body fat increased by 0.20 [0.13–0.27] and 0.65 [0.59–0.72] % at Visits 2 and 3, respectively, compared with Visit 1.

Changes in continuous variables between Visits 1–2 and Visits 2–3 were subjected to a pair-wise comparison (Table 2). In general, there was an increase in some metabolic indices but no increase in obesity since social distancing was implemented. The increases in skeletal muscle and fat mass, BP, pulse, glycated hemoglobin, and serum levels of total cholesterol and LDL cholesterol during the Visit 2–3 period were larger than those in the Visit 1–2 period, although there was no additional notable increase in body weight, BMI, WC, serum levels of glucose, triglycerides, and HDL cholesterol during the Visit 2–3 period.

**Table 2:** Changes in obesity indices and metabolic markers at each visit.

**N = 7,875**	**Visit 2–Visit 1**	**Visit 3–Visit 2**	***P*-value**
Δ Body weight (kg)	0.16 ± 2.68	0.16 ± 2.78	0.878
Δ BMI (kg/m^2^)	0.07 ± 0.92	0.04 ± 0.98	0.047
Δ Waist circumference (cm)	0.47 ± 4.50	−0.50 ± 4.79	<0.001
Δ Skeletal muscle mass (kg)	0.07 ± 1.39	0.46 ± 2.03	<0.001
Δ Fat mass (kg)	0.19 ± 2.20	0.35 ± 2.44	<0.001
Δ Fat percentage (%)	0.23 ± 2.51	0.40 ± 2.79	<0.001
Δ Blood pressure (mmHg)			
Systolic	0.80 ± 11.21	1.48 ± 11.32	<0.001
Diastolic	−0.29 ± 8.41	0.96 ± 8.53	<0.001
Δ Pulse (/min)	0.01 ± 8.78	0.97 ± 8.70	<0.001
Δ Glucose (mg/dL)	1.10 ± 10.59	0.43 ± 11.03	<0.001
Δ HbA1c (%)	−0.00 ± 0.29	0.06 ± 0.30	<0.001
Δ Total cholesterol (mg/dL)	0.03 ± 26.60	2.15 ± 27.83	<0.001
Δ Triglyceride (mg/dL)	1.42 ± 70.67	−0.58 ± 68.05	0.070
Δ HDL cholesterol (mg/dL)	0.72 ± 7.93	0.59 ± 7.90	0.309
Δ LDL cholesterol (mg/dL)	−0.01 ± 23.43	0.83 ± 24.47	0.028
Δ Non-HDL cholesterol (mg/dL)	−0.69 ± 25.59	1.56 ± 26.69	<0.001

Data are expressed as the mean ± standard deviation.

P-values are calculated using paired t-test.

BMI, body mass index; HbA1c, glycated hemoglobin; HDL, high density lipoprotein; LDL, low density lipoprotein.

### Odd ratios for metabolic syndrome and metabolic comorbidities at visits 2 and 3

**Figure 3:**
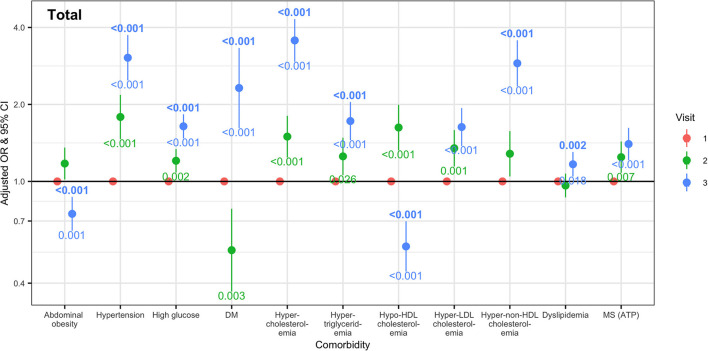
Odd ratios and 95% confidence intervals for metabolic comorbidities at Visits 2 and 3 compared with Visit 1. Odd ratios and confidential intervals were calculated using a generalized linear mixed model, adjusted according to sex, age group, smoking, and alcohol drinking. The numbers above and below the odd ratios and confidential interval bars at Visit 2 and 3 are the result of *post-hoc* analysis using the Bonferroni method. DM, diabetes mellitus; HDL, high density lipoprotein; LDL, low density lipoprotein; MS, metabolic syndrome.

Considering changes in medication for hypertension, diabetes, and dyslipidemia, we calculated the odds ratios for metabolic syndrome and its components, and categorized metabolic comorbidities at Visit 2 and 3 according to the definitions described in the Methods (measurement); this was done to ascertain whether the odds increased significantly at Visit 3. The calculated odds ratios and *post-hoc* test results are shown in Figure 3. The odds of hypertension, high glucose, and diabetes were higher at Visit 3. Regarding cholesterol abnormalities, while the odds of hypo-HDL cholesterolemia decreased at Visit 3, those of hypercholesterolemia, hypertriglycerides, hyper-non-HDL cholesterolemia, and dyslipidemia increased at Visit 3. The odds of hyper-LDL cholesterolemia and metabolic syndrome, as defined by NCEP–ATP III, were already increased before the social distancing.

### Prevalence of metabolic complications at each visit according to age group and health check-up center location

The prevalence of metabolic complications, i.e., metabolic syndrome, high BP, hypertension, diabetes, hypercholesterolemia, elevated serum glucose, or dyslipidemia, was calculated and compared among the three visits; subjects were subgrouped according to age and health check-up center location (Table 3). The prevalence of metabolic complications at Visit 3 was high in the group aged 40–59 attending health check-up centers located in the capital area.

**Table 3:** Prevalence of metabolic complications at each visit according to age group and health check-up center location.

			**Area**									
			**Capital**					**Non-capital**				
		**Metabolic complications** ^**a**^	**Visit 1**	**Visit 2**	**Visit 3**	***P*** **-for trend**	***P*** **-value**	**Visit 1**	**Visit 2**	**Visit 3**	***P*** **-for trend**	***P*** **-value**
Age	−39	N	1,413	1,152	913			299	264	196		
		Metabolic syndrome	232 (17.13)	191 (16.81)	150 (17.58)	0.824	0.903	47 (15.77)	56 (21.29)	35 (17.95)	0.421	0.238
		High BP	276 (20.23)	218 (19.17)	204 (23.69)	0.085	0.039	91 (30.43)	102 (38.64)	75 (38.27)	0.052	0.077
		High serum glucose	247 (18.19)	222 (19.51)	228 (26.48)	0	<0.001	71 (23.83)	68 (25.86)	37 (18.97)	0.279	0.218
		Hypertension	103 (7.57)	97 (8.54)	97 (11.32)	0.003	0.009	41 (13.71)	48 (18.18)	42 (21.43)	0.023	0.075
		Diabetes mellitus	24 (2.05)	18 (1.83)	13 (1.81)	0.685	0.907	10 (3.46)	9 (3.52)	8 (4.19)	0.695	0.906
		Hypercholesterolemia	140 (10.29)	109 (9.58)	102 (11.92)	0.298	0.232	20 (6.71)	18 (6.84)	21 (10.77)	0.124	0.201
		Dyslipidemia	341 (24.89)	279 (24.52)	253 (29.08)	0.043	0.040	67 (22.48)	75 (28.52)	50 (25.64)	0.336	0.260
	40–59	N	4,434	4,661	4,820			1,298	1,311	1,342		
		Metabolic syndrome	897 (22.33)	1,025 (23.43)	1,020 (25.60)	0.001	0.002	275 (21.24)	292 (22.32)	287 (21.42)	0.915	0.770
		High BP	1,376 (33.52)	1,471 (33.30)	1,654 (40.08)	<0.001	<0.001	528 (40.71)	568 (43.39)	617 (45.98)	0.006	0.024
		High serum glucose	1,196 (29.36)	1,430 (32.38)	1,740 (42.18)	<0.001	<0.001	420 (32.36)	431 (32.90)	358 (26.68)	0.001	<0.001
		Hypertension	831 (20.53)	935 (21.37)	975 (24.41)	<0.001	<0.001	295 (22.78)	312 (23.83)	351 (26.15)	0.043	0.117
		Diabetes mellitus	292 (8.10)	326 (8.26)	371 (10.32)	0.001	<0.001	117 (9.14)	123 (9.47)	132 (9.91)	0.502	0.797
		Hypercholesterolemia	846 (20.91)	932 (21.29)	1062 (26.76)	<0.001	<0.001	183 (14.11)	213 (16.26)	231 (17.23)	0.029	0.082
		Dyslipidemia	1,616 (39.17)	1,713 (38.47)	1,834 (43.59)	<0.001	<0.001	462 (35.62)	460 (35.11)	470 (35.07)	0.770	0.948
	60-	N	282	316	396			149	171	208		
		Metabolic syndrome	83 (32.55)	97 (33.11)	101 (30.79)	0.629	0.812	39 (26.35)	40 (23.53)	43 (20.67)	0.209	0.454
		High BP	148 (56.92)	184 (60.73)	217 (62.90)	0.141	0.328	76 (51.35)	94 (55.29)	116 (55.77)	0.428	0.682
		High serum glucose	116 (44.44)	140 (46.98)	181 (52.92)	0.035	0.097	64 (42.95)	71 (41.52)	76 (36.54)	0.207	0.418
		Hypertension	121 (47.27)	151 (51.36)	158 (48.47)	0.826	0.607	59 (39.86)	62 (36.47)	87 (41.83)	0.640	0.568
		Diabetes mellitus	45 (20.00)	50 (19.16)	65 (21.89)	0.564	0.714	20 (14.29)	22 (13.10)	26 (12.68)	0.674	0.909
		Hypercholesterolemia	102 (39.84)	127 (43.94)	148 (46.69)	0.102	0.259	29 (19.46)	45 (26.32)	61 (29.33)	0.038	0.105
		Dyslipidemia	140 (52.63)	166 (55.70)	194 (57.74)	0.213	0.456	58 (38.93)	69 (40.35)	89 (42.79)	0.456	0.753

Data are expressed as N (%). P-values for trend are calculated using Cochrane-Armitage test for trend and p-values using Chi-squared test.

^a^High BP was defined as systolic BP ≥130 mmHg or diastolic BP ≥ 85 mmHg, or taking hypertension drugs. High serum glucose was defined as fasting glucose ≥100 mg/dL or taking diabetes drugs. Hypertension was defined as systolic BP ≥140 mmHg or diastolic BP ≥ 90 mmHg, or taking hypertension drugs. Diabetes mellitus was defined as fasting glucose ≥126 mg/dL or ≥ 6.5%, or taking diabetes drugs. Hypercholesterolemia was defined as serum total cholesterol ≥ 240 mg/dL, or taking dyslipidemia drugs. Dyslipidemia was defined as having hypercholesterolemia, serum triglyceride ≥200 mg/dL, serum HDL cholesterol <40 mg/dL, serum LDL cholesterol ≥160 mg/dL, or taking dyslipidemia drugs.

BP, blood pressure; HDL, high density lipoprotein; LDL, low density lipoprotein.

## Discussion

This retrospective cohort study conducted using large health check-up data from Korean adults in university hospitals between January 2018 and June 2021 shows that the odds of metabolic complications, including hypertension, high glucose, diabetes, hypercholesterolemia, hypertriglyceridemia, hyper-non-HDL-cholesterolemia, and dyslipidemia, were elevated markedly since implementation of high level social distancing measures to deal with the third wave of COVID-19; however, there were only small but steady increases in anthropometric indices and body composition. In addition, the increase in metabolic complications induced by high level social distancing were particularly prevelant in relatively young adults who visited health check-up centers located in the capital area. Previous studies report inconsistent results regarding the effects of COVID-19 on obesity and its associated metabolic comorbidities. However, our study is different from previous ones that made simple comparisons before and after COVID-19. As mentioned in the introduction, we first selected examinees after implementation of systematic social distancing measures and obtained retrospective data for these people. Therefore, it was possible to check the effect of social distancing on obesity and its metabolic comorbidities objectively, and to compare data before and after social distancing. It is a reliable study design, and the results confirm the impact of social distancing more clearly.

In this study, we found that body weight, BMI, and body fat percentage showed a modest and gradual increase, whereas WC decreased, during social distancing in Korea. The amount of increase through Visits 1, 2, and 3, in body weight, BMI, and body fat percentage are more likely due to aging and the general increase in obesity in Korea, not significantly affected by COVID-19 and social distancing. This result is different from those of previous studies showing prominent weight gain and worsening of obesity during the pandemic and associated social distancing measures and/or lockdowns. There may be several reasons for this discrepancy. First, previous studies enrolled a relatively small number of subjects, and based their analysis not on measured data but on answers to questionnaires (12–18), which are subject to recall bias. Here, we enrolled nearly 8,000 subjects from multiple health check-up centers and used directly measured data regarding anthropometry and body composition. Second, the study design and follow-up period differed from those of previous studies. We used a retrospective cohort design with a longer follow-up period and repeatedly measured data. Because the duration between Visits 1–2 and Visits 2–3 was more than 1 year, our data does not reflect acute change in body weight caused by COVID-19 and social distancing. Third, the characteristics of the study population were different from those in previous studies. The population in the present study is older than those in previous studies (13, 14, 17, 18). In addition, the socioeconomic status of our participants is higher than that of the general population; such participants checked their health status regularly, even during the pandemic. This means that they were employees of companies that provided regular health check-ups, or were prosperous and had a strong interest in their own health. Fourth, although social distancing might worsen metabolic parameters, it is possible that isolation for just a few months may not be enough to affect body composition or weight.

As mentioned, although a significant increase in BMI and WC was not observed since the implementation of social distancing, most metabolic parameters (except hypo-HDL-cholesterolemia) worsened. A cross-sectional study conducted in Turkey reported no significant deterioration in body weight gain or glycemic control in type 2 DM patients during the national lockdown (21). Also, another observational study analyzing healthy adults found no significant change in body weight during COVID-19 lockdown, although serum glucose, total cholesterol, and LDL cholesterol increased significantly after lockdown was lifted (22).

While there was no data regarding participants' eating habits and daily activities, we suggest that lifestyle changes induced by COVID-19 and the related social distancing measures might be the root of the metabolic derangement. These lifestyle changes can be categorized as greater consumption of low quality food, increased meal frequency, and sedentary behavior. Previous studies reported lifestyle changes during the COVID-19 pandemic. For example, more poor quality foods were being consumed; indeed, consumption of unhealthy snacks/desserts and sugar-sweetened beverages increased during the early phase of the pandemic (23). Consumption of stockpiled foods such as instant noodles and frozen foods also increased. Meal frequency and snacking, snacking at night, and consumption of comfort foods such as chocolate, ice cream, and desserts increased during confinement (24). According to a survey conducted in Seoul in 2020, online purchases and food delivery increased during the COVID-19 outbreak, and this increase in consumption was especially prevalent among younger people (11). Behavioral restrictions to suppress transmission of COVID-19 also decreased physical activities. Studies that measured physical activity objectively using accelometers or pedometers reported that the number of steps (25–27) and duration of physical activity decreased (8, 26, 28). Regarding these changes in lifestyle pattern, blood metabolic indices most likely deteriorated in the group most likely to be affected by restricted activities and the eating environment imposed by social distancing.

Total, HDL, LDL cholesterol, and triglycerides increased during social distancing. However, the increase of HDL cholesterol might be secondary to increases in total serum lipids. Non-HDL cholesterol comprises all atherogenic lipoproteins, including LDL, IDL, VLDL cholesterol, and lipoprotein(a) (29). The degree of increase between Visit 2 and 3 was more prominent for non-HDL cholesterol than for HDL cholesterol (Table 2). Therefore, while increased HDL cholesterol tends to prevent cardiovascular events, the overall change in the lipid profile of this cohort during social distancing worsened.

However, the changes of lifestyle induced by COVID-19 and related lockdowns or social distancing were not always undesirable, nor were they the same for diverse subgroups of the population. The pandemic decreased consumption of food away from the home and increased the amount of home cooking (24, 30). With respect to physical activity, younger people and people living in urban areas were affected more severely by lockdown (9, 28). A study in the UK found that spatial and socioeconomic status were linked to decreased mobility during the early stages of the COVID-19 pandemic (31). This suggests that COVID-19 and related social distancing have different effects on people living in different areas. Another notable finding of this study is that the metabolic derangement was most marked in those aged 40–59 years attending health check-up in centers located in the capital area. In South Korea, the capital area is the most densely populated region, with the highest number of confirmed COVID-19 infections (2). As such, it is the area that had either the same or more strict social distancing measures, with more severe restriction of various activities.

This study has several limitations. First, selection bias cannot be ruled out. The participants were limited to “university hospital health check-up examinees.” Subjects were individuals who visited the hospital regularly for health check-ups or had support from their workplaces, even during the COVID-19 pandemic; thus they were more concerned about their health, lived a relatively healthy life style, and were likely of higher and more stable socioeconomic status than the general population. These participants do not represent the general Korean population. We tried to overcome this limitation by gathering a large number of subjects from diverse areas of South Korea. Second, there were differences in equipment and tools used for blood tests and body composition measurement among the health check-up centers. Moreover, body composition analysis was performed using the bioelectrical impedance analysis method. While body composition analysis with bioelectrical impedance analysis can be performed easily without exposure to radiation, there are limitations in that the test is affected by the amount of biological body fluid. Therefore, we excluded subjects who underwent procedures or had diseases that may affect their amount of body fluid, such colonoscopy or chronic kidney disease. Third, there was no information about diet and physical activity levels; therefore, we could not evaluate the effect of high level social distancing on the participants' lifestyle.

However, a strength of this study is that it included a large number of people from various areas with major cities in South Korea, and it is very meaningful that the analysis was based on the results of patient tests conducted at the hospital, unlike previous that used questionnaires and self-reporting. In addition, we analyzed data continuously from before the COVID-19 pandemic until the introduction of high level social distancing measures. In other words, we conducted a more accurate and detailed analysis, making it possible to compare, analyze, and follow-up changes in individuals from before the start of COVID-19 pandemic to the middle of the pandemic and after implementation of high level social distancing.

In conclusion, this retrospective cohort study shows that the odds of metabolic syndrome and its components increased after implementation of high level social distancing measures, although there were no clear changes in body weight, WC, or body composition. Additionally, the increased odds of metabolic complications was most noticeable in relatively young adults who visited health check-up centers located in the capital area. We suggest the need for a focused approach for prevention and management of metabolic worsening in subpopulations who are prone to be more sedentary and to eat unhealthy food during the COVID-19 pandemic and enforced social distancing. Healthcare workers should check and manage weight changes and metabolic parameters in patients at high risk.

## Data availability statement

The datasets analyzed in this study are not readily available because they were participants' personal health information. Requests to access the datasets should be directed to the corresponding author followed by approvals from institutional review boards.

## Ethics statement

The studies involving human participants were reviewed and approved by Institutional Review Boards of Kyungpook National University Hospital (KNUH 2021-08-011), Daegu Catholic University Medical Center (CR-21-134), Gachon University Gil Medical Center (GDIRB2021-313), Konyang University Hospital (KYUH 2021-08-023), CHA Bundang Medical Center (CHAMC 2021-08-047), Chaum Life Center (CHAMC 2021-08-047), Inje University Paik Hospital (PAIK 2021-09-008), Jeju National University Hospital (JEJUNUH 2021-08-016), Chung-Ang University Hospital (CAUH 2108-026-19381), Wonju Severance Christian Hospital (CR321095), Inje University Ilsan Paik Hospital (ISPAIK 2021-08-040), Korea University Guro Hospital (2021GR0466), and International St. Mary's Hospital (21YeonIRB068). Written informed consent for participation was not required for this study in accordance with the national legislation and the institutional requirements.

## Author contributions

H-JK and YC: conceptualization, investigation, writing–original draft preparation, and project administration. K-KK: conceptualization, project administration, investigation, formal analysis, visualization, writing–review and editing, and supervision. J-HK, Y-SK, J-HH, and Y-IH: project administration, data collection, and data interpretation. H-IC, KL, JP, SC, J-KK, TL, M-JS, YY, YS, GN, and SK: data collection and data interpretation. All authors critically revised the manuscript for important intellectual content and approved the final version.

## Funding

The Study of Obesity and Metabolic Syndrome provided the data and fund (SOMS 2021-001) for this study in 2021.

## Conflict of interest

The authors declare that the research was conducted in the absence of any commercial or financial relationships that could be construed as a potential conflict of interest.

## Publisher's note

All claims expressed in this article are solely those of the authors and do not necessarily represent those of their affiliated organizations, or those of the publisher, the editors and the reviewers. Any product that may be evaluated in this article, or claim that may be made by its manufacturer, is not guaranteed or endorsed by the publisher.
