# Supplementary material for: COVID-19 and related social distancing measures induce significant metabolic complications without prominent weight gain in Korean adults
Source: Front Med (Lausanne). 2022 Sep 7;9:951793. doi: 10.3389/fmed.2022.951793 (PMC9490037; doi:10.3389/fmed.2022.951793)
Supplement: Supplementary file 1 [file Table_1.DOCX]

**Supplementary table. The changes in medication at each visit**

|  | Visit 1 | Visit 2 | Visit 3 | P−Value | P for trend |
| --- | --- | --- | --- | --- | --- |
| N | 7875 | 7875 | 7875 |  |  |
| Antihypertensives | 920 (12.5) | 1003 (13.3) | 967 (14.1) | 0.015 | 0.004 |
| Anti-diabetic drugs | 307 (4.2) | 347 (4.6) | 344 (5.0) | 0.049 | 0.014 |
| Anti-dyslipidemic drugs | 687 (9.3) | 763 (10. 2) | 812 (11.9) | < 0.001 | < 0.001 |

Data are expressed as N (%). P-values are calculated using the Chi-squared test and p-values for trend (P for trend) are calculated using Cochrane-Armitage test for trend and P-values are calculated using the Chi-squared test.
