# Supplementary figures and images for: COVID-19 and related social distancing measures induce significant metabolic complications without prominent weight gain in Korean adults
Source: Front Med (Lausanne). 2022 Sep 7;9:951793. doi: 10.3389/fmed.2022.951793 (PMC9490037; doi:10.3389/fmed.2022.951793)

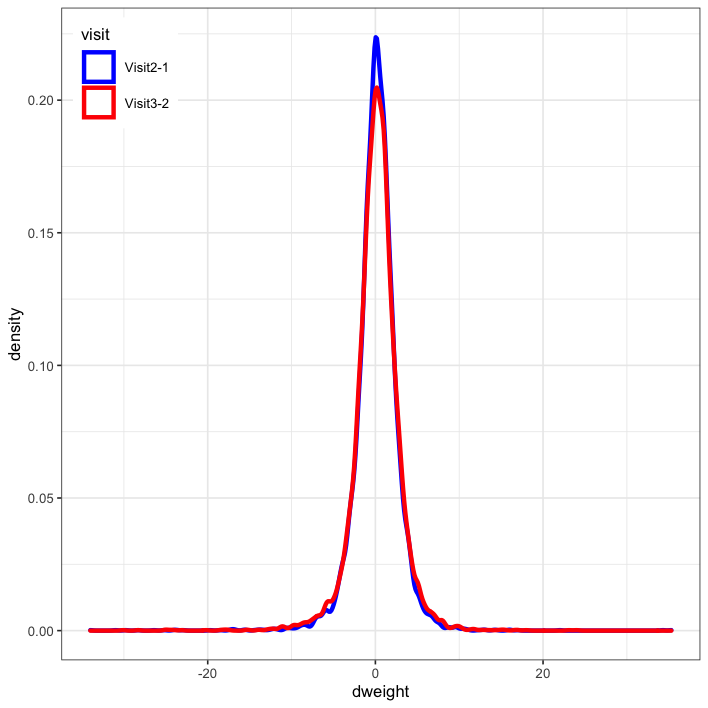

Supplement: Supplementary file 2 [file Data_Sheet_1.ZIP › Supplement_Figure_1.tiff]

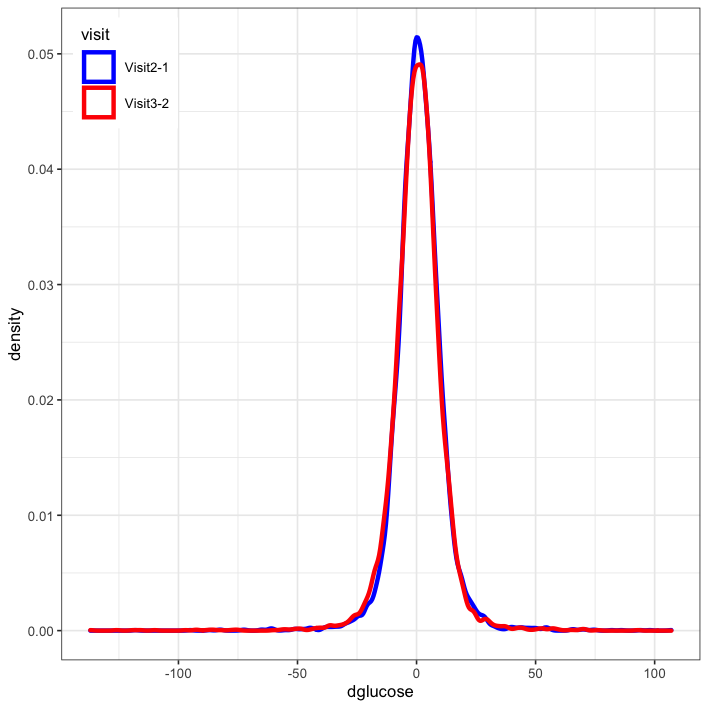

Supplement: Supplementary file 2 [file Data_Sheet_1.ZIP › Supplement_Figure_10.tiff]

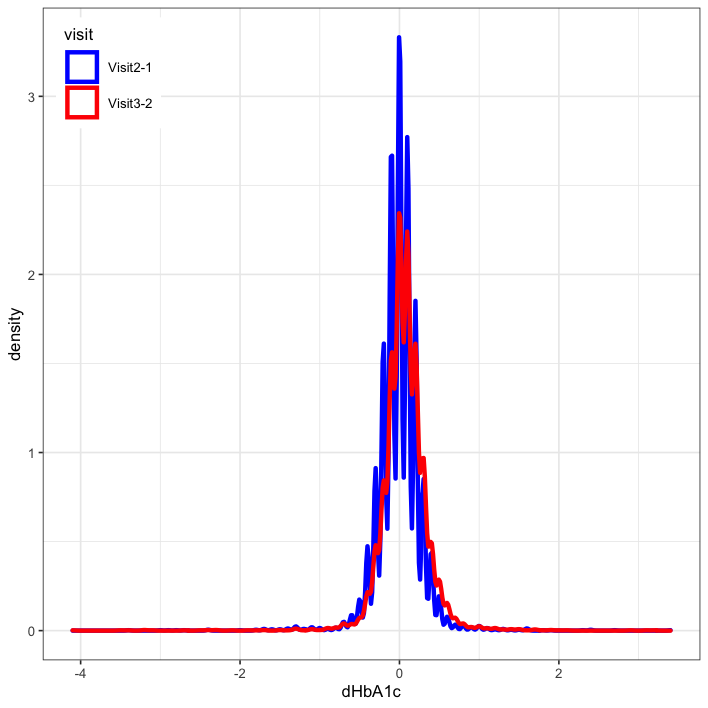

Supplement: Supplementary file 2 [file Data_Sheet_1.ZIP › Supplement_Figure_11.tiff]

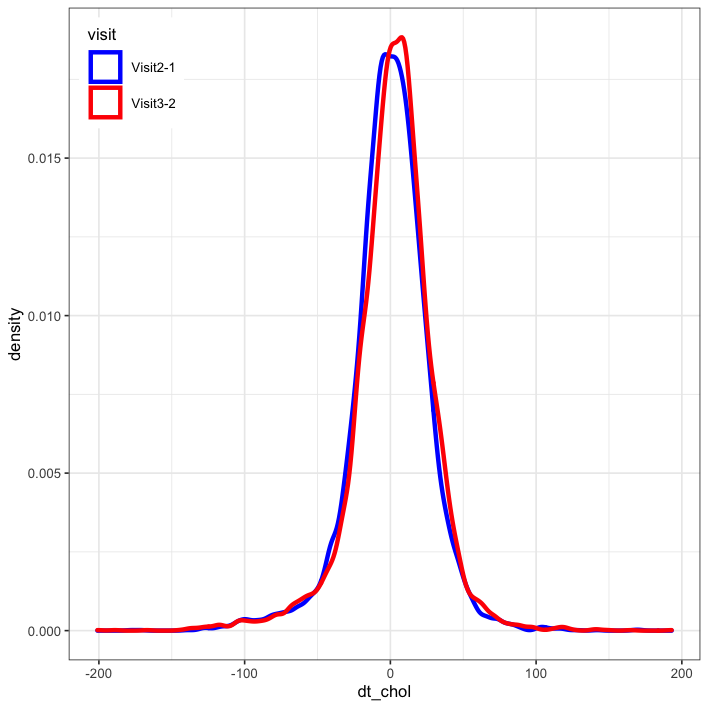

Supplement: Supplementary file 2 [file Data_Sheet_1.ZIP › Supplement_Figure_12.tiff]

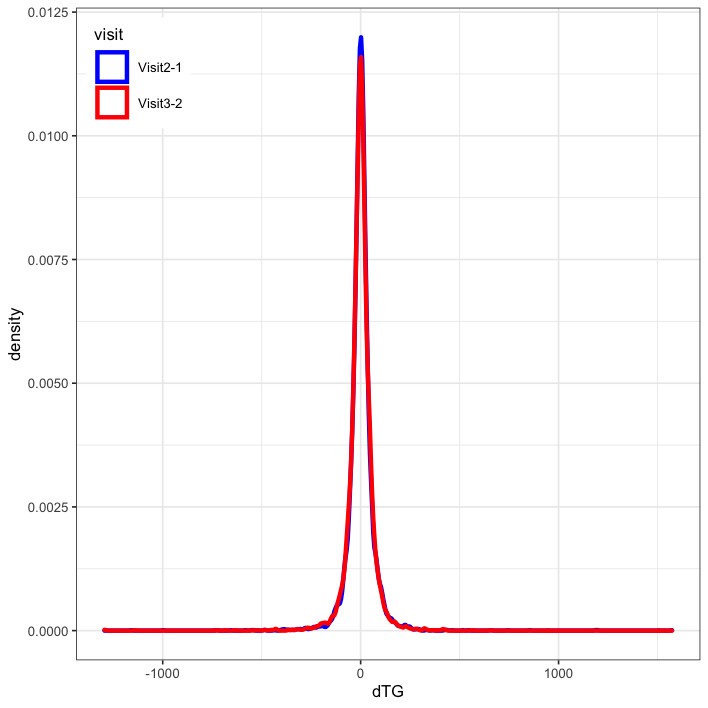

Supplement: Supplementary file 2 [file Data_Sheet_1.ZIP › Supplement_Figure_13.tiff]

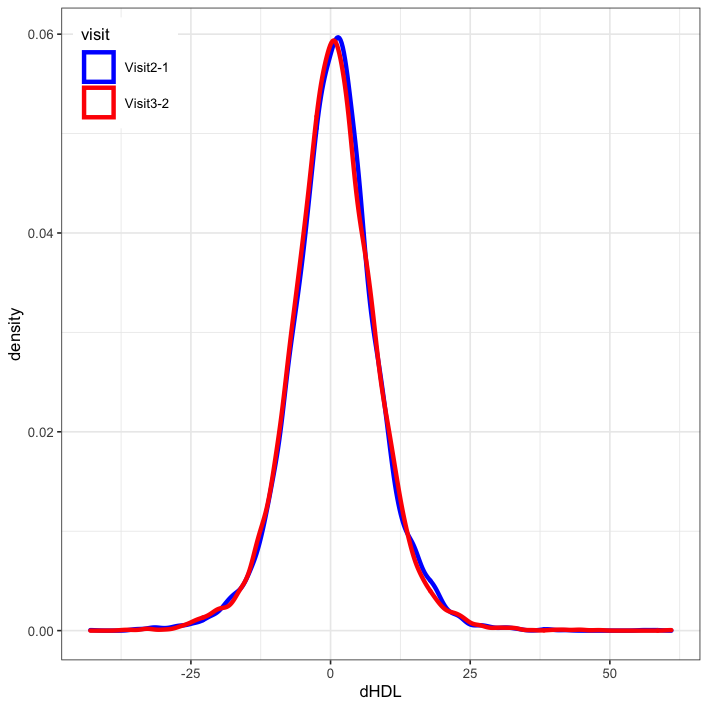

Supplement: Supplementary file 2 [file Data_Sheet_1.ZIP › Supplement_Figure_14.tiff]

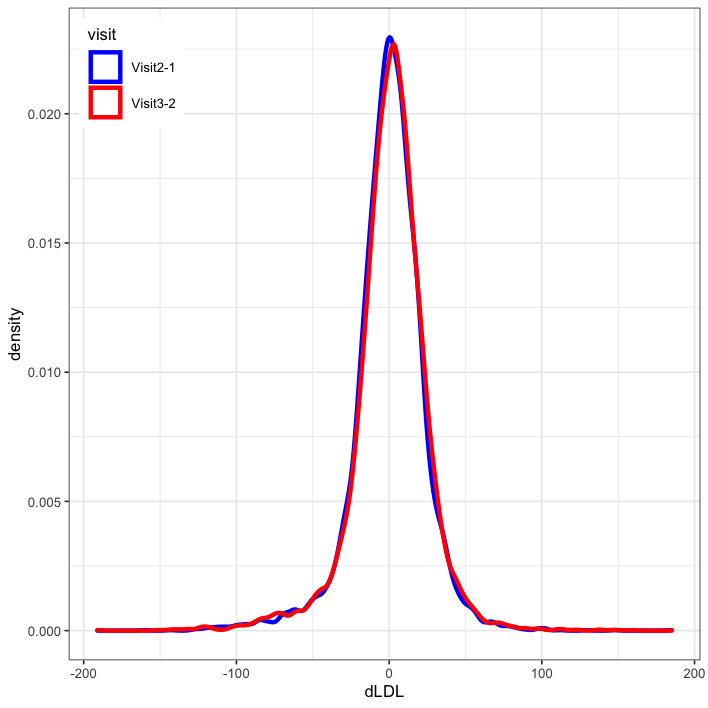

Supplement: Supplementary file 2 [file Data_Sheet_1.ZIP › Supplement_Figure_15.tiff]

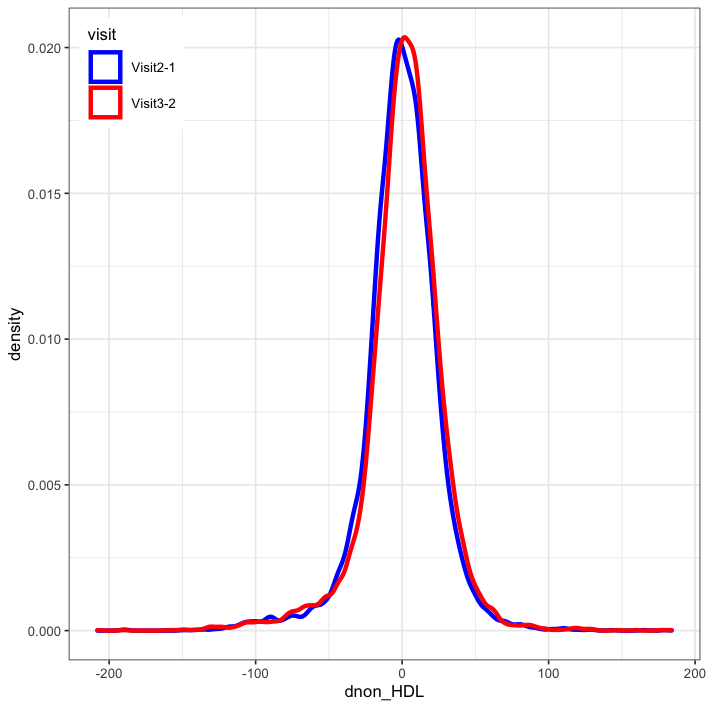

Supplement: Supplementary file 2 [file Data_Sheet_1.ZIP › Supplement_Figure_16.tiff]

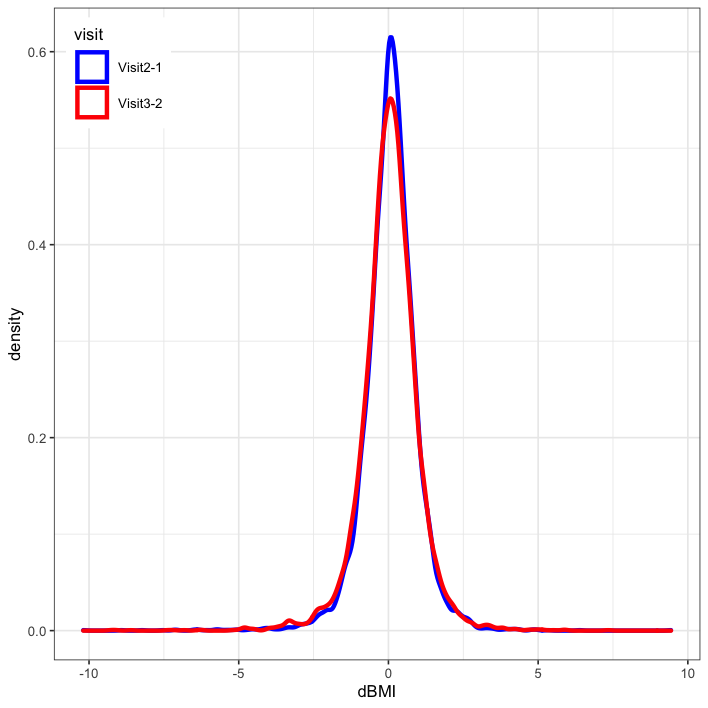

Supplement: Supplementary file 2 [file Data_Sheet_1.ZIP › Supplement_Figure_2.tiff]

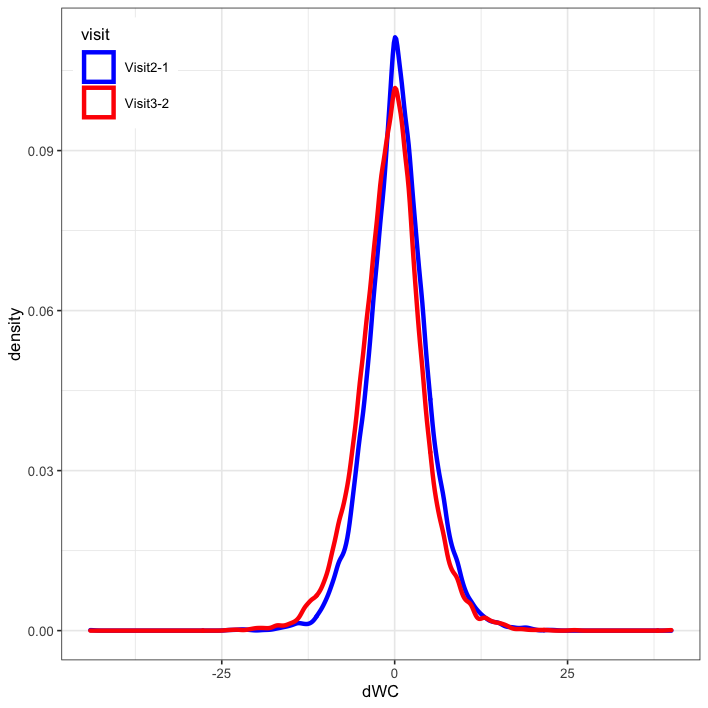

Supplement: Supplementary file 2 [file Data_Sheet_1.ZIP › Supplement_Figure_3.tiff]

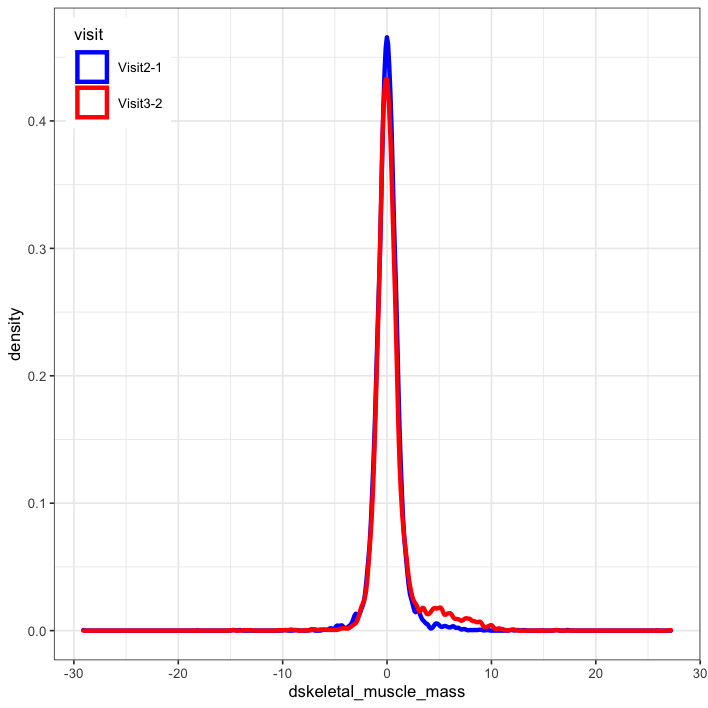

Supplement: Supplementary file 2 [file Data_Sheet_1.ZIP › Supplement_Figure_4.tiff]

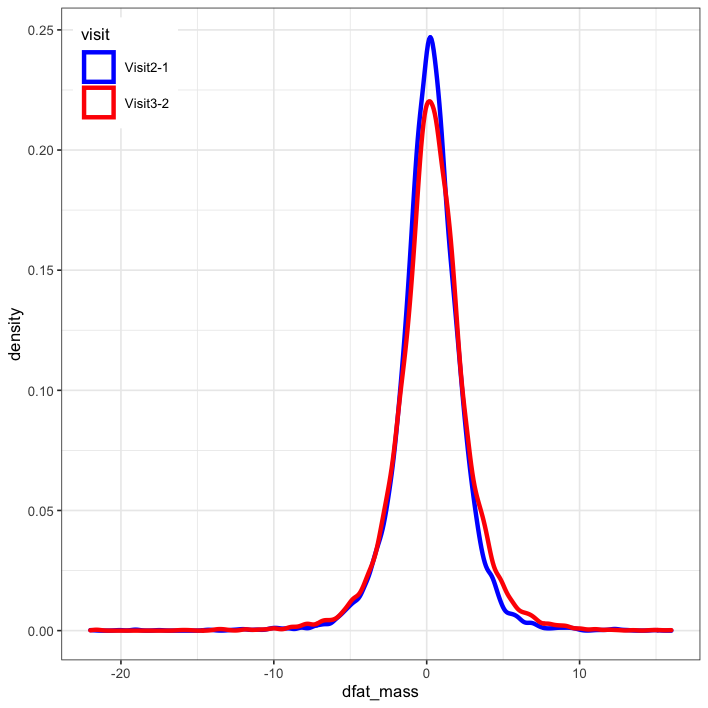

Supplement: Supplementary file 2 [file Data_Sheet_1.ZIP › Supplement_Figure_5.tiff]

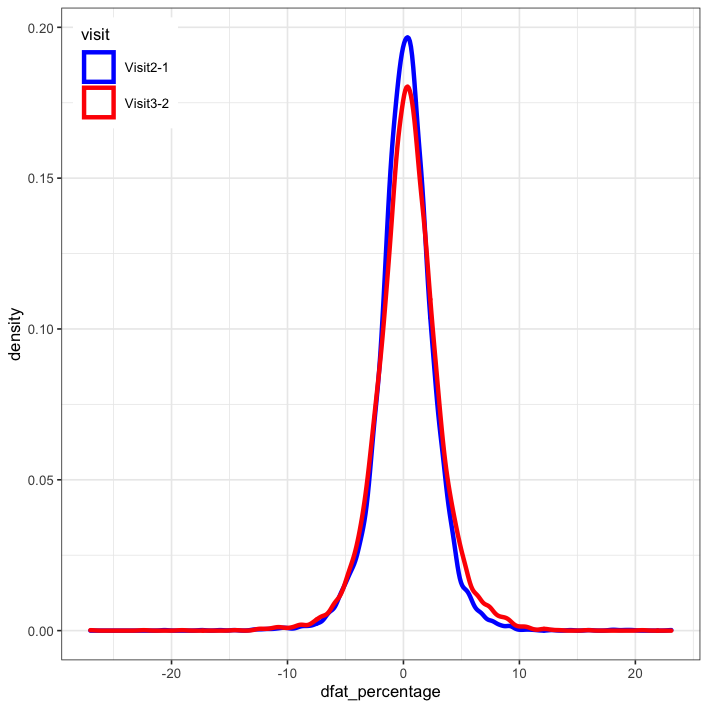

Supplement: Supplementary file 2 [file Data_Sheet_1.ZIP › Supplement_Figure_6.tiff]

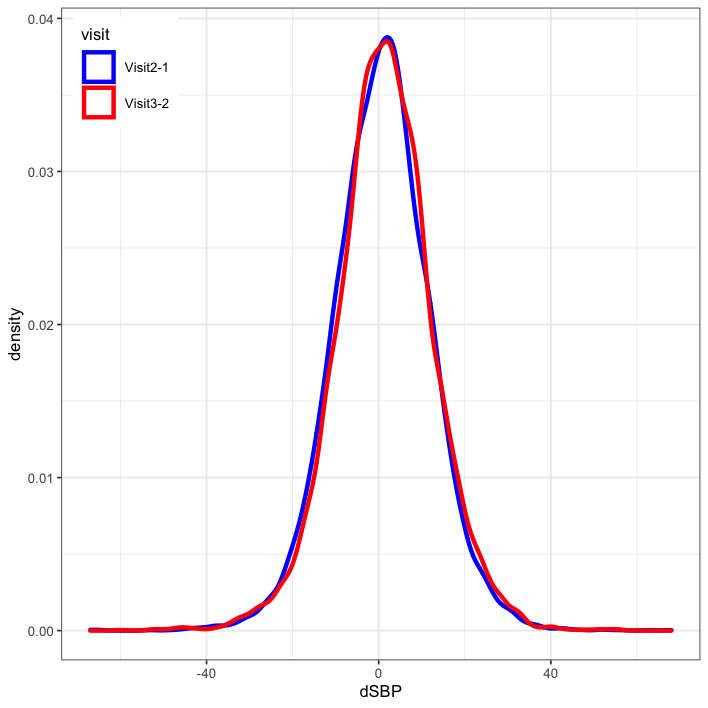

Supplement: Supplementary file 2 [file Data_Sheet_1.ZIP › Supplement_Figure_7.tiff]

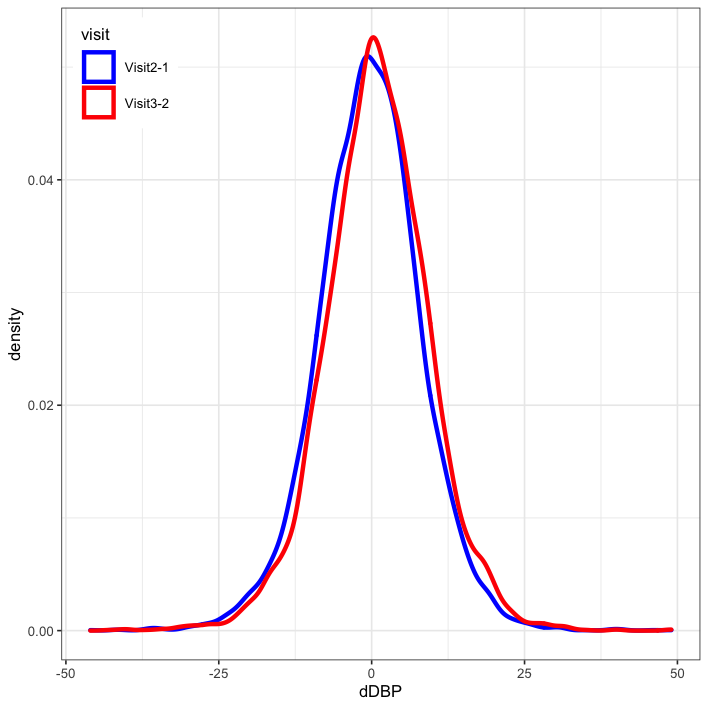

Supplement: Supplementary file 2 [file Data_Sheet_1.ZIP › Supplement_Figure_8.tiff]

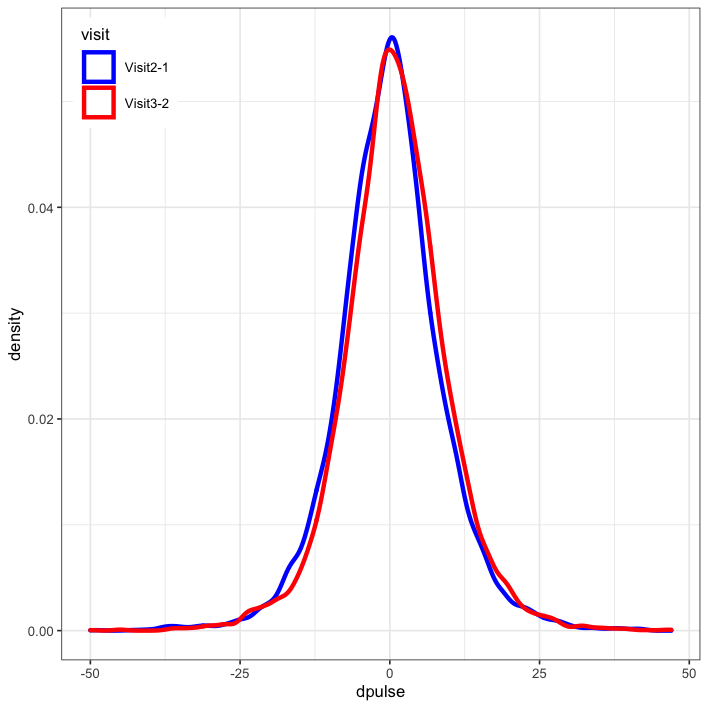

Supplement: Supplementary file 2 [file Data_Sheet_1.ZIP › Supplement_Figure_9.tiff]
